# Reduction of cellular stress is essential for Fibroblast growth factor 1 treatment for diabetic nephropathy

**DOI:** 10.1111/jcmm.13921

**Published:** 2018-10-15

**Authors:** Yanqing Wu, Yiyang Li, Ting Jiang, Yuan Yuan, Rui Li, Zeping Xu, Xingfeng Zhong, Gaili Jia, Yanlong Liu, Ling Xie, Ke Xu, Hongyu Zhang, Xiaokun Li, Jian Xiao

**Affiliations:** ^1^ Institute of Life Sciences Wenzhou University Wenzhou Zhejiang China; ^2^ Molecular Pharmacology Research Center School of Pharmaceutical Science Wenzhou Medical University Wenzhou Zhejiang China; ^3^ The First Clinical Medical College Gannan Medical University Ganzhou Jiangxi China; ^4^ Department of Anesthesiology Second Affiliated Hospital of Wenzhou Medical College Wenzhou China

**Keywords:** diabetes nephropathy (DN), endoplasmic reticulum (ER) stress, fibroblast growth factor 1 (FGF1), fibrosis, oxidative stress

## Abstract

Diabetic nephropathy (DN) is one of general and common complication of diabetes, which severely affects the physical and mental health of diabetic patients. Fibroblast growth factor 1 (FGF1), an effective control agent of blood glucose, plays an effective treatment role on diabetes‐induced renal injury. But the specific molecule mechanism underlying it is still unclear. Since induction of cellular stress is the main and common mechanism of diabetes‐induced complication, we hypothesized that reduction of cellular stress is also the molecular mechanism of FGF1 treatment for DN. Here, we have further confirmed that FGF1 significantly ameliorated the diabetes‐induced renal interstitial fibrosis and glomerular damage. The expression levels of collagen and α‐smooth muscle actin (α‐SMA) also dramatically induced in kidney from db/db mice, but these effects were blocked by FGF1 administration. Our mechanistic investigation had further revealed that diabetes significantly induced oxidative stress, nitrosative stress, and endoplasmic reticulum (ER) stress with upregulation of malondialdehyde (MDA), nitrotyrosine level, ER stress makers and downregulation of antioxidant capacity (AOC). FGF1 treatment significantly attenuated the effect of diabetes on cellular stress. We conclude that FGF1‐associated glucose decreases and subsequent reduction of cellular stress is the another potential molecule mechanism underlying FGF1 treatment for DN.

## 1 INTRODUCTION

Diabetes mellitus (DM) is one kind of lifelong metabolic diseases characterized by multiple causes of hyperglycemia, leading to a series of complications with diabetic retinopathy, diabetic neuropathy, and diabetic nephropathy (DN).^1^ DN is one of the most common and serious complications of diabetic patients. The patho‐mechanism of DN is multifactorial and complicated. Free fatty acids, advanced glycated end products (AGEs), autophagy, and angiotensin II receptor pathway are known to be participated in the development of DN.^2^ With a rising prevalence of diabetes, it is greatly important to illuminate molecular mechanism underlying DN and seek the proper and effective treatment strategies to reduce patient morbidty and financial burden due to DN.

Cellular stress is a component of the development of various kidney diseases.^3^, ^4^ Our mechanism studies have revealed that elevated oxidative stress and ER stress are the causal events for diabetes‐related complications.^5^, ^6^ Oxidative stress is a condition in which generation of reactive oxygen species (ROS) exceeds the capacity of the antioxidant defense system, caused by increased ROS production and impaired antioxidant capacity.^7^ Subsequent studies have also implicated that the NADPH oxidases (NOX) family of enzymes as major cytosolic sources of superoxide, and it is now appreciated that several sources exist within the cell that contribute to the increased oxidative stress accompanying diabetes.^4^ It has been demonstrated hyperglycemia induces oxidative and nitrosative stress, and increases renal functional impairment via nuclear factor erythroid‐2 related factor 2 (Nrf2) signalling.^8^, ^9^ Evidence suggests that ER stress not only contributes to the pathogenesis of acute kidney diseases, but also has a role in the progression to chronic kidney diseases. Treatment with 4‐PBA (an ER stress inhibitor) attenuates the increases of renal ER stress markers, α‐smooth muscle actin (α‐SMA), connective tissue growth factor, tubulointerstitial fibrosis and apoptosis.^3^, ^10^

Fibroblast growth factor 1 (FGF1) is an autocrine/paracrine regulator and known to act on cells from a variety of tissue origins including the liver, vasculature, and skin, exerting classic mitogenic activity 11 and neuroprotective role.^12^ Our previous study had demonstrated that FGF1 treatment ameliorated diabetes‐induced nephropathy by inhibiting inflammation via JNK/NF‐kB signalling pathway.^13^ But there are no studies to focus on the effect of FGF1 on cellular stress during FGF1 treatment for DN. It is well known that hyperglycemia resulting in glucotoxicity is the main factor for diabetes‐induced complications.^5^, ^6^ As an insulin sensitizer, FGF1 is also capable of impinging on multiple pathways mediating homeostatic control of normal glycemia.^14^, ^15^ FGF1 reduces the level of oxidative stress in high glucose treated H9c2 cells and HMVECs.^16^ Our previous study had also demonstrated that FGF1 treatment blocks ER stress and consequently apoptosis of DA neuron during development of Parkinson's disease (PD).^17^ Moreover, FGF23, the FGF family, has also an anti‐oxidative stress activity.^18^, ^19^ Thus, we hypothesized that FGF1‐associated glucose decreases and subsequent reduction of cellular stress is the another potential cellular molecule mechanisms mediating FGF1 treatment forDN.

In present study, we had further confirmed the protective role of FGF1 on DN with attenuation of renal fibrosis and glomerulosclerosis. Moreover, it was found that FGF1 administration blocked diabetes‐induced oxidative stress though NOX2‐ROS‐Nrf2 signalling, and elevated ER stress evidencing with induction of ER stress makers in kidney. Current study suggests that reduction of cellular stress is the another potential molecular mechanism underlying FGF1 treatment for DN, which further replenishes the molecular mechanism underlying FGF1 treatment for DN.

## 2 MATERIALS AND METHODS

### 2.1 Animal and experimental design

12‐week old male db/db (C57BLKS/J‐leprdb/leprdb) mice and their nondiabetic db/m littermates were purchased from the Model Animal Research Center of Nanjing University (Nanjing, China). The animals were maintained under a 14‐h light/10‐h dark condition. After arrived, the animals were acclimatized to animal house before use. The db/db mice were divided into two groups and intraperitoneally (i.p.) injected either with FGF1 (0.5 mg/kg body weight) or physiologic saline every other day for 4 weeks. After 4 weeks, blood glucose level and body weight were measure. The serum was used for detection the levels of MDA (Beyotime, Shanghai, China), SOD (Beyotime) and AOC (Beyotime) using assay kits. The kidneys from mice were collected for biochemical and molecular analyses.

### 2.2 H&E staining and immunohistochemistry

The kidneys were collected and fixed with 4% paraformaldehyde in phosphate‐buffered saline (PBS). Kidneys were dehydrated in alcohol and embedded with paraffin. After that, 5‐μm sections were dewaxed and hydrated, then stained with hematoxylin and eosin (H&E) and observed under light microscope. After dewaxing and hydration, the sections were also incubated in 3% H_2_O_2_ for 15 minutes and then in blocking solution for 45 minutes. Subsequently, the sections were incubated at 4°C over‐night with the following primary antibodies: α‐SMA (1:500; Abcam, Cambridge, UK), Collagen I (1:500; Abcam), Collagen III (1:500; Abcam), SOD2 (1:500; Santa Cruz Biotechnology, Santa Cruz, CA, USA) and HO‐1 (1:200; Santa Cruz Biotechnology). After washing in PBS three times, the sections were incubated with horseradish peroxidase‐conjugated secondary antibodies for 2 hours at 37°C. Then, the sections were reacted with 3, 3‐diaminobenzidine (DAB). The results were imaged using a Nikon ECLPSE 80i (Nikon, Tokyo, Japan).

### 2.3 Immunofluorescence staining

The kidneys were collected and fixed with 4% paraformaldehyde in phosphate‐buffered saline (PBS). Kidneys were dehydrated in alcohol and embedded with paraffin. After dewaxing, rehydrating and washing in PBS, the 5‐μm sections were respectively incubated with 5% bovine serum albumin (BSA) in 37°C oven for 0.5 hours. Then, the sections were incubated with mouse anti‐IgM (1:400; Abcam) as primary antibody in 4°C overnight. After triple washing in PBS at room temperature, the sections were once again incubated with donkey anti‐mouse TR (1:1000; Abcam) as secondary antibody for 4 h. Fluorescence images were captured using a Nikon ECLPSE 80i.

### 2.4 Masson staining and PAS staining

Briefly, the kidneys were collected and fixed with 4% paraformaldehyde in phosphate‐buffered saline (PBS). After dewaxing, rehydrating, and washing in PBS, 5‐μm thick paraffin‐embedded cardiac tissue sections were stained using standard masson trichrome staining kits (Solarbio Science & Technology, Beijing, China, G1340) and PAS staining kits (Solarbio Science & Technology, G1280) according to the manufacturer's instructions. Digital pictures were captured using a Nikon ECLPSE 80i.

### 2.5 Immunoblotting

Immunoblotting was performed as previously described. Briefly, the kidneys from different experimental groups were sonicated in the lysis buffer, containing a protease inhibitor cocktail (Sigma, St Louis, MO). Equal amounts of protein and the Precision Plus Protein Standards (Bio‐Rad, Hercules, CA, USA) were resolved by SDS‐PAGE electrophoresis and transferred onto PVDF membranes. Membranes were incubated in 5% nonfat milk for 45 min and then incubated for 18 hours at 4°C with the primary antibodies at dilution of 1:1000 in 5% non‐fat milk. To test whether equivalent amounts of protein were loaded among all samples, membranes were stripped and incubated with a mouse antibody against GADPH (Abcam) to generate a signal used as a loading control. Using the Super Signal West Femto Maximum Sensitivity Substrate kit (Thermo Scientific, Rockford, IL, USA), chemiluminescence emitted from the bands was directly captured using a UVP Bioimage EC3 system. Densitometric analysis of chemiluminescence signals was performed using VisionWorks LS software. The quantitative analysis of protein was calculated as the densitometric value of phosphorylated protein level/the densitometric value of unphosphorylated protein. All experiments were repeated in triplicate with the use of independently prepared tissue lysates.

### 2.6 Dihydroethidium (DHE) staining

Dihydroethidium staining was used to detect superoxide. DHE reacts with superoxide that is bound to cellular components including protein and DNA and exhibits bright red fluorescence. The kidneys were fixed in 4% paraformaldehyde (PFA) for 30 minutes, washed three times with PBS (5 minutes per wash), and then embedded in OCT. 10‐mm frozen embryonic sections were incubated with 1.5 mmol L^−1^ DHE for 5 minutes at room temperature, and then washed three times with PBS for 5 minutes per wash. Sections were counterstained with DAPI and mounted with aqueous mounting medium (Sigma).

### 2.7 Statistical analyses

Data were presented as means ± SEM. Experiments were repeated at least three times, and kidneys from each replicate were from different mice. Statistical differences were determined by one‐way analysis of variance (ANOVA) using SigmaStat 3.5 software. In one‐way ANOVA analysis, Turkey test was used to estimate the significance of the results (*P* < 0.05).

## 3 RESULTS

### 3.1 FGF1 treatment ameliorated diabetes‐induced renal injury

**Figure 1:**
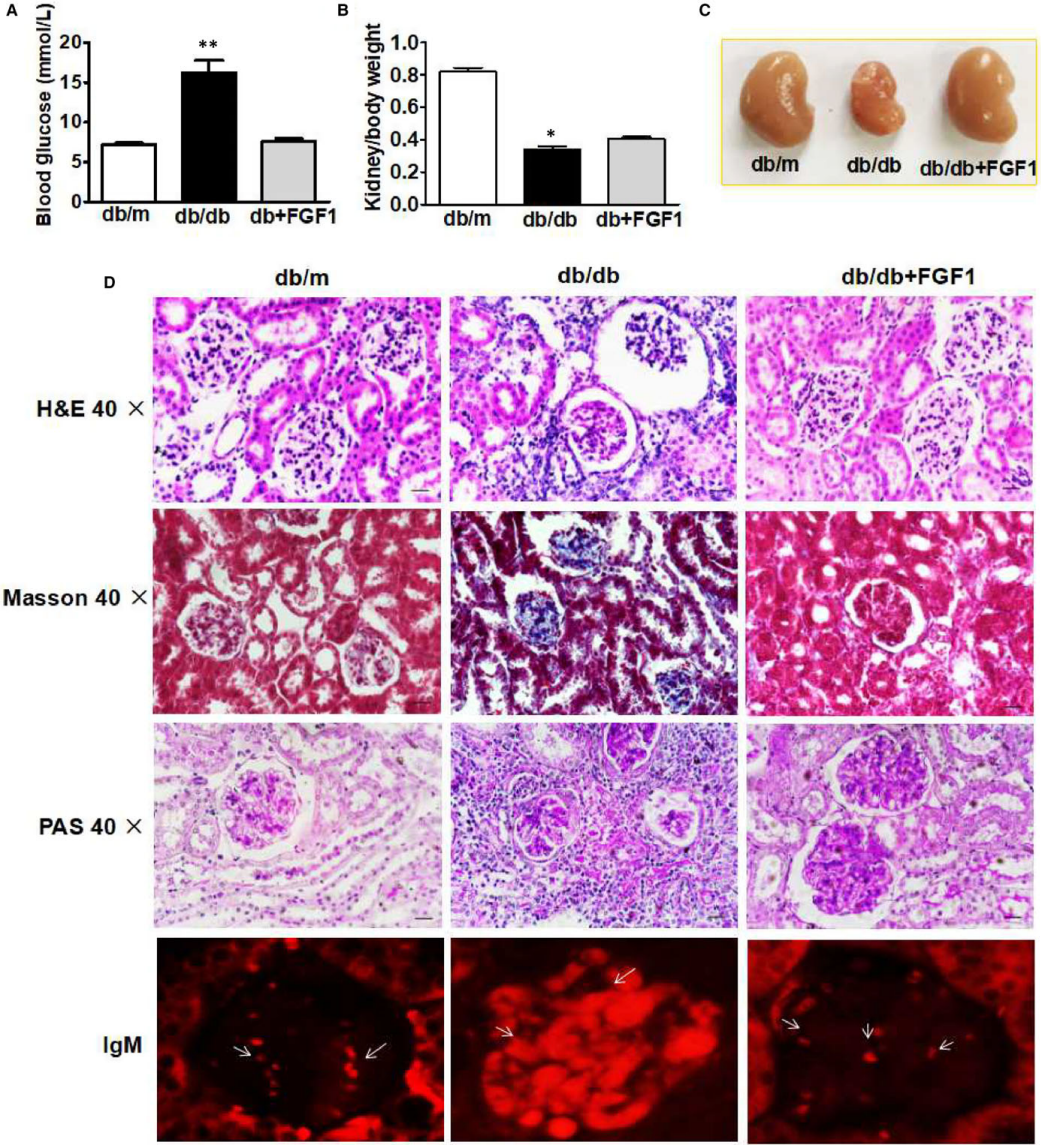
FGF1 treatment ameliorated diabetes‐induced renal injury. A, Blood glucose level of mice from db/m, db/db and db+FGF1 group after fasting for 16 h; B, The ratio of kidney/body weight of mice from db/m, db/db and db+FGF1 group; C, Morphological appearance of kidney from db/m, db/db and db+FGF1 group; D, The H&E, Masson staining, PAS staining and IgM staining of kidney from db/m, db/db and db+FGF1 group. All data are presented as the mean ± SEM, n = 3. *P < 0.05 and **P < 0.01 vs the db/m group and db+FGF1 group

Here, we had confirmed that FGF1 significantly ameliorated diabetes‐induced nephropathy. We found that FGF1 treatment significantly downregulated the blood glucose level of db/db mice (Figure 1A). The ratio of kidney/body weight in db/db miceis significant lower than that in db/m mice with remarkable nephrarctia in db/db group, which is reversed by FGF1 treatment (Figure 1B,C). Morphological analysis showed significant renal interstitial fibrosis and glomerular damage in kidneys from db/db mice as indicated by glomerular mesangial expansion with hypercellularity, capillary collapse, and fibrous deposition in glomeruli and interstitial, which is significantly attenuated by FGF1 treatment (Figure 1D). Additionally, the IgM was dispersively deposited in glomerular mesangium in db/db mice's kidney. FGF1 treatment significantly blocked IgM deposition in kidney (Figure 1D). Taken together, above data further confirmed that FGF1 treatment ameliorated diabetes‐induced renal dysfunction.

### 3.2 FGF1 treatment remitted diabetes‐induced renal interstitial fibrosis

**Figure 2:**
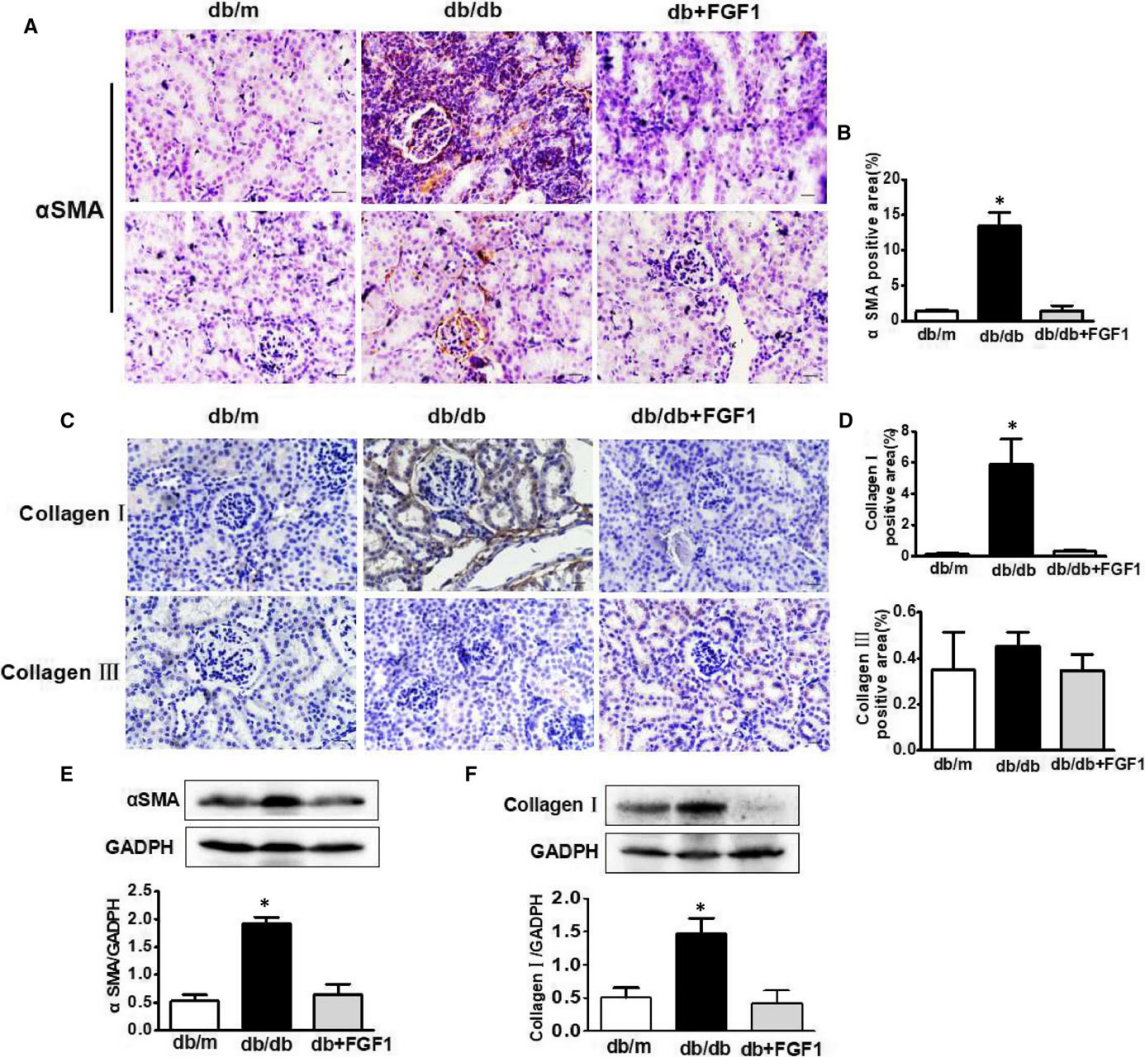
FGF1 treatment remitted diabetes‐induced renal interstitial fibrosis. A‐D, Immunohistochemical staining of α‐SMA, collagen I and collagen III of kidney from db/m, db/db and db+FGF1 group (scale bars = 30 μm); E and F, The protein expression of α‐SMA and collagen I of kidney from db/m, db/db and db+FGF1 group. All data are presented as the mean ± SEM, n = 3. *P < 0.05 vs the db/m group and db+FGF1 group

The expression levels of collagen and α‐SMA in the outer medulla was used as the index of interstitial injury. We found that the positive staining areas of α‐SMA and collagen I were significantly larger in the kidneys from db/db mice, but not collagen III, which were reversed by FGF1 treatment (Figure 2A‐D). Consistence with the immunohistochemical results, further quantitation of collagen I and α‐SMA expression in kidneys by Western blot analyses also showed that the protein levels of α‐SMA and collagen I in kidneys from db/db mice were significantly upregulated, but much lower in the FGF1 treatment group (Figure 2E,F). These results suggested that FGF1 treatment remitted diabetes‐induced renal interstitial fibrosis by abolishing the increases of collagen accumulation and α‐SMA expressions in kidneys.

### 3.3 FGF1 treatment reduced diabetes‐induced oxidative stress and nitrosative stress in kidney

**Figure 3:**
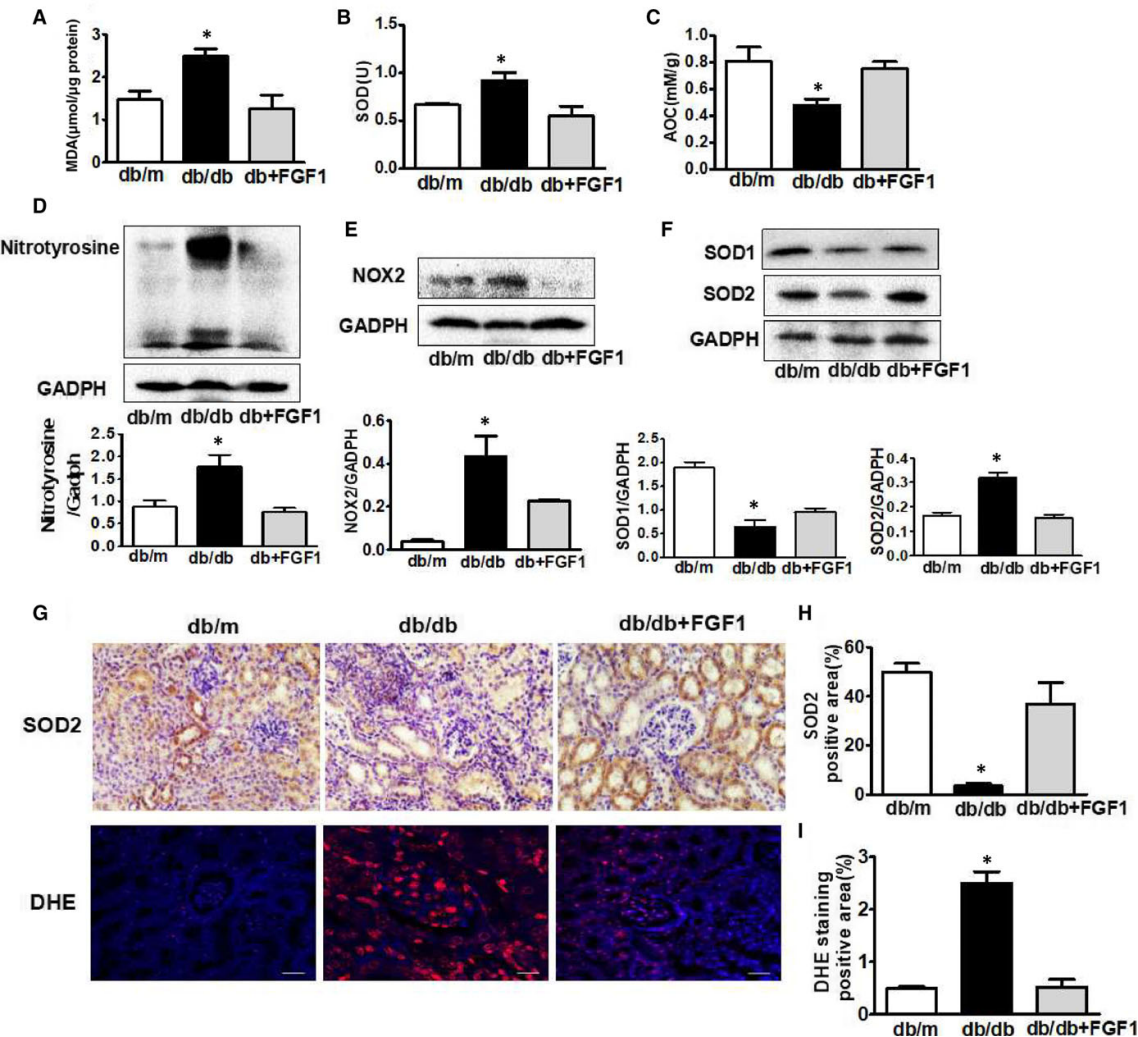
FGF1 treatment reduced diabetes‐induced oxidative stress and nitrosative stress in kidney. A‐C, The levels of MDA, SOD and AOC of serum from db/m, db/db and db+FGF1 group; D‐F, The protein expression of nitrotryosine, NOX2, SOD1 and SOD2 of kidney from db/m, db/db and db+FGF1 group; G and H, Immunohistochemical staining of SOD2 of kidney from db/m, db/db and db+FGF1 group (scale bars = 30 μm); G and I, DHE staining of kidney from db/m, db/db and db+FGF1 group (scale bars = 30 μm). All data are presented as the mean ± SEM, n = 3. *P < 0.05 vs the db/m group and db+FGF1 group

**Figure 4:**
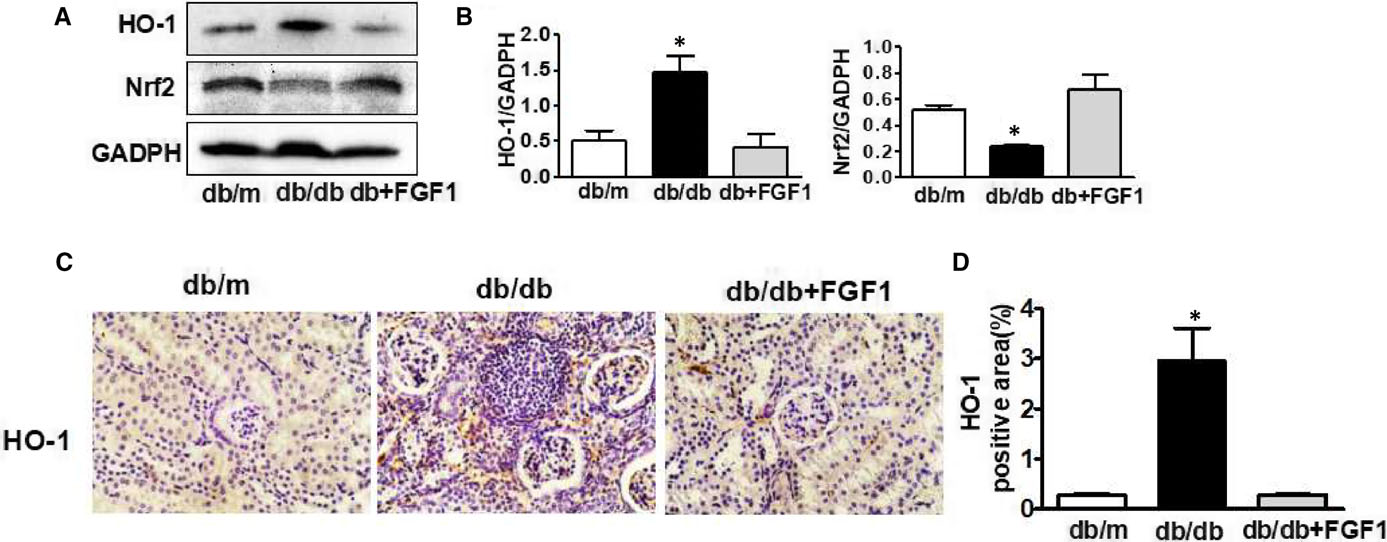
The effect of Nrf2/HO‐1 signalling during FGF1administration for diabetes‐induced oxidative stress and nitrosative stress in kidney. A and B, The protein expression of HO‐1 and Nrf2 of kidney from db/m, db/db and db+FGF1 group; C and D, Immunohistochemical staining of HO‐1of kidney from db/m, db/db and db+FGF1 group (scale bars = 30 μm). All data are presented as the mean ± SEM, n = 3. *P < 0.05 vs the db/m group and db+FGF1 group

Oxidative stress is the major molecular mechanism that involves in diabetic complication.^20^, ^21^ Here, we try to determine whether FGF1 treatment reverses diabetes‐induced oxidative stress and subsequently ameliorates renal dysfunction. Oxidative stress makers and nitrosative stress makers were detected and presented in Figure 3. It was found that lipid peroxidation (LPO) (MDA/mg protein), antioxidant biomarker (superoxide dismutase, SOD) in serum was significantly upregulated in kidney from db/db group (Figure 3A,B), and the total antioxidant capacity (AOC) levels in diabetic kidney was significantly less than those in control group (Figure 3C). Although the SOD level in serum was upregulated, SOD1 and SOD2 expression levels in kidney were significantly downregulated in db/db mice when compared with those in db/m group and FGF1 treatment group (Figure 3F‐H). Elevated level of nitrotyrosine modified protein is indicative of nitrosative stress. Level of nitrotyrosine modified protein in diabetic kidney was significantly higher than that in control group (Figure 3D). NOX activity is increased in different animal models of renal injury, which participates in the generation of superoxide and/or hydrogen peroxide. Here, we found that FGF1 administration significantly suppressed diabetes‐induced overexpression of NOX2 in kidney (Figure 3E). The positive signalling of DHE staining in kidney from db/db mice is much strong than that in db/m group and FGF1 treatment group (Figure 3G,I). Moreover, FGF1 treatment suppressed diabetes‐induced down‐regulation of Nrf2 expression (Figure 4A,B). However, the expression level of HO‐1 level was upregulated (Figure 4A‐D). Taken together, FGF1 treatment reversed diabetes‐induced oxidative stress and nitrosative stress in kidney.

### 3.4 FGF1 treatment suppressed diabetes‐induced ER stress and up‐regulation of Bax in kidney

**Figure 5:**
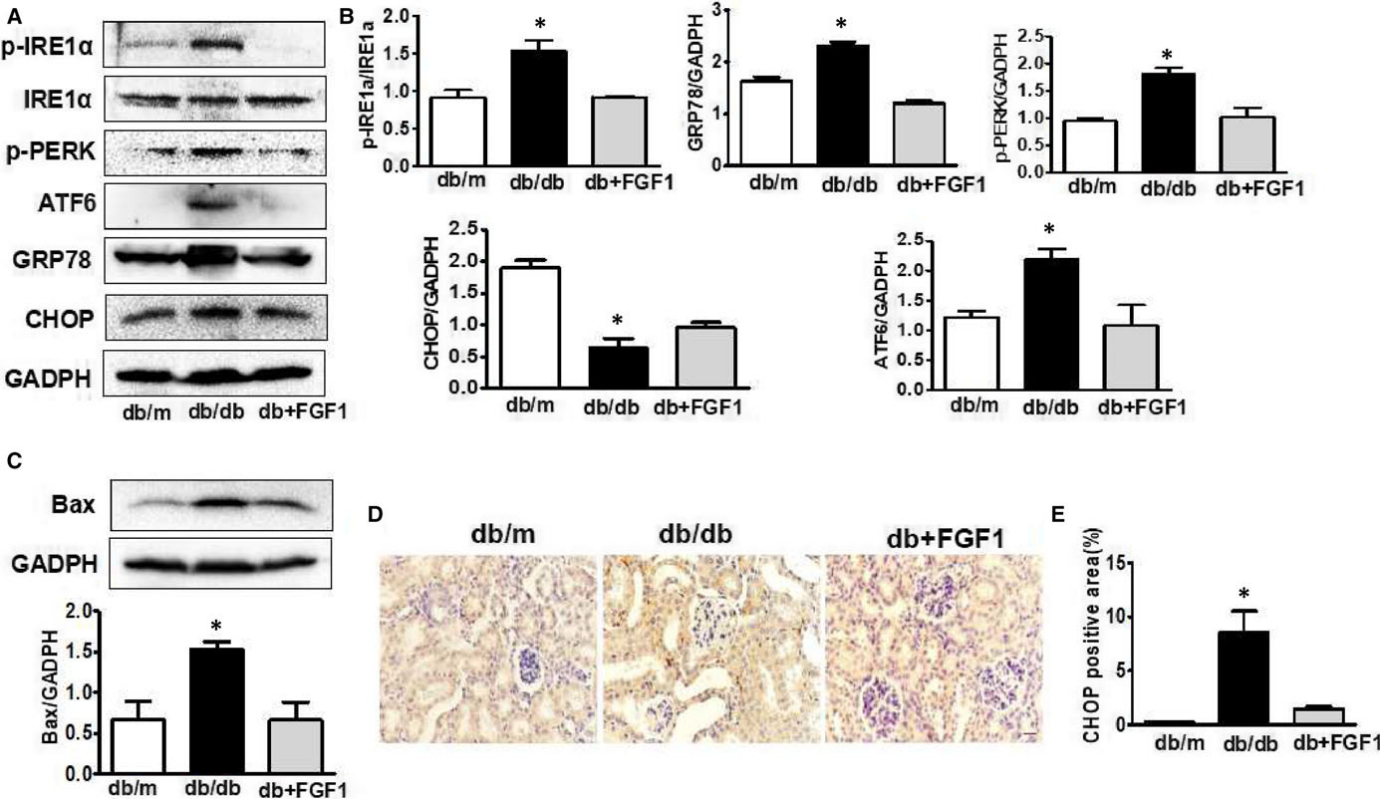
FGF1 treatment suppressed diabetes‐induced ER stress and up‐regulation of Bax in kidney. A, The protein expression of p‐PERK, p‐IRE1α, ATF6, GRP78 and CHOP of kidney from db/m, db/db and db+FGF1 group; B, Intensities of p‐IRE1α normalized to IRE1α, p‐PERK, ATF6, CHOP and GRP78 normalized to GADPH; C, The protein expression of Bax of kidney from db/m, db/db and db+FGF1 group; D and E, Immunohistochemical staining of CHOP of kidney from db/m, db/db and db+FGF1 group (scale bars = 30 μm). All data are presented as the mean ± SEM, n = 3. *P < 0.05 vs the db/m group and db+FGF1 group

To detect whether FGF1 treatment inhibited diabetes‐induced ER stress in kidney, we had detected the expression levels of ER stress makers. The protein levels of phosphorylated protein kinase RNA‐like ER kinase (p‐PERK), phosphorylated inositol‐requiring protein‐1α (p‐IRE1α), activating transcription factor 6 (ATF6), glucose regulated protein 78 (GRP78)and C/EBP‐homologous protein(CHOP) in kidney were significantly induced by diabetes (Figure 5A,B). FGF1 treatment significantly suppressed diabetes‐induced ER stress (Figure 5A,B). Consistence with Western blotting results, the fluorescence intensity of CHOP was up‐regulated in kidney from db/db mice (Figure 5D,E). Moreover, elevated expression level of Bax was observed in kidneys from db/db mice (Figure 5C). Our current results suggest that ER stress may be involved in the FGF1 administration‐mediated attenuation of DN.

## 4 DISCUSSION

DN is a serious and common complication of diabetes, which leads to end‐stage kidney disease,^4^, ^22^ and therefore, it is important to illuminate the mechanisms and explore the effective therapeutic strategy for DN. Specific treatment of patients with diabetic nephropathy can be divided into 4 major arenas: cardiovascular risk reduction, glycemic control, blood pressure control, and inhibition of the renin‐angiotensin system (RAS).^1^ Our previous studies have demonstrated that FGF1 administration protected against renal injury by reducing inflammation.^13^ Based on previous study, our current study had further confirmed that FGF1 administration ameliorated diabetes‐induced glomerular damage and interstitial fibrosis. Mechanistic studies had found that the induction of cellular stress in diabetic kidneys was blocked by FGF1 treatment, indicating that reduction of cellular stress is another potential crucial molecular mechanism during FGF1 treatment for DN.

Our current study had demonstrated that FGF1 treatment blocked diabetes‐associated α‐SMA overexpression and collagen accumulation. Histologically, progressive kidney disease is marked by renal fibrosis and glomerulosclerosis.^4^, ^23^ It is well known that α‐SMA is the marker of cell transdifferentiation, including epithelial–mesenchymal transition (EMT) and fibroblast activation into myofibroblast, which importantly contributes to the progression of CKD.^24^, ^25^ Our results suggest that FGF1 treatment may be involved in the relatively early stage of pathogenic process of DN via ameliorating tubulointerstitial damage.

Our current study had verified the important regulatory effect of FGF1 for cellular stress during DN. Previous studies had reported that FGF1 suppressed the oxidative stress and consequently blocked diabetes‐induced cardiomyopathy.^16^ Our current study has found that FGF1 treatment blocked the diabetes‐induced oxidative stress during DN. It was widely regarded that elevated cellular stress is a major causal event of diabetes‐associated the onset of complications.^6^, ^26^ Diabetes significantly induces oxidative stress 27 and ER stress.^28^ Hemodialysis and CKD patients manifest impaired mitochondrial respiration indicative of dysregulated aerobic metabolism and increased oxidative stress.^29^ NADPH oxidases (NOX) family has been significantly induced during renal injury, and reduced NOX activity is associated with renal protection in pre‐clinical models of CKD.^30^, ^31^ Nox1, Nox2, and Nox4, which are expressed in both human and rodent kidneys, have a central role in mediating oxidative stress in CKD.^32^ These studies suggest that oxidative stress, involving various reactive oxygen and nitrogen species, exerts a promoted role in the progress of CKD development.^4^ Consistence with prior studies, we found that diabetes significantly triggered oxidative stress evidencing with the upregulated expression of nitrotryosine andNOX2, and reduction of SODs expression and AOC activity in kidney from db/db mice. FGF1 treatment blocked the dysfunction of oxidability and antioxidant ability in kidney.

Nrf2 signalling regulates expression of many genes that oppose inflammatory and oxidative damage, including HO‐1, SODs, and NQO1, which is protective in various models of renal disease. In current study, diabetes significant induced down‐regulation of Nrf2 expression and SODs, but the expression level of HO‐1 level was upregulated. It was well known that the Nrf2 signalling may compensatorily increase for responsing to acute stress. In our study, the mice were under hyperglycemia condition for a long time, which exceeds the compensatory capacity and leads to the downregulation of Nrf2 and SODs. We speculate that the expression of HO‐1 will also decrease as time gone on. These data indicated that FGF1 treatment ameliorated hyperglycemia and subsequently oxidative stress during DN via NOX2‐ROS‐Nrf2 signalling.

Multiple studies have shown that induction of ER stress are responsible for the pathogenetic progression of DN.^3^, ^33^ ER stress occurs when misfolded proteins accumulate in the ER lumen and cause ER dysfunction, which is known as unfolded protein response (UPR).^34^, ^35^ The UPR is mediated by the activation of three major sensors: IRE1, PERK and ATF6, which are suppressed by binding to GRP78. Upon ER stress is activated, GRP78 is released from the combination of IRE1, PERK and ATF6 to bind accumulated dysfunctional proteins.^36^ Our data are consistent with the prior finding where it was shown that diabetes significantly triggered ER stress markers expression in kidney, which was blocked by FGF1treatment.

FGF1, is an autocrine/paracrine regulator whose binding to heparan sulphate proteoglycans, plays a role on proliferation, neuroprotection and effectively normalizing hyperglycemia in T2D mice. Previous studies had found that pharmacologically relevant FGF1 doses (0.5 mg/kg) to T2D mouse models (ie, ob/ob or db/db) with impaired insulin sensitivity led to impressive changes in several measures of metabolism in which blood glucose was nearly normalized and long‐lasting (35 days).^37^ Our results expanded on this notion by showing that FGF1 blocked the hyperglycemia in db/db mice. Although type 2 diabetes is a complex metabolic disorder, hyperglycemia with resulting glucotoxicity is a major mediator of diabetes‐induced complications. The kidney is the main target organ involved in the major complications caused by diabetes mellitus.^1^, ^38^ Based on above results, we can conclude that FGF1 normalized the metabolic activity and subsequently blocked cellular stress, finally ameliorated kidney complications in diabetes.

An interesting finding in present study was verified that reduction of oxidative stress and ER stress contributes to the FGF1 treatment for DN. However, there are some limitations in present study. It has been postulated that ER stress is involved in redox homeostasis via activation of Nrf2.^8^ But the causal relationship between oxidative stress and ER stress during DN is still unknown. Moreover, although our and other's studies have demonstrated that both cellular stress and inflammation are the potential mechanism contributing for FGF1 treatment for DN, whether cellular stress is mutually regulated with inflammation during DN still isn't elucidated. The present study did not further provide the evidence of the relationship between cellular stress and inflammation. Therefore, it is interesting to further explore the causal relationship between oxidative stress and ER stress, cellular stress, and inflammation during DN.

**Figure 6:**
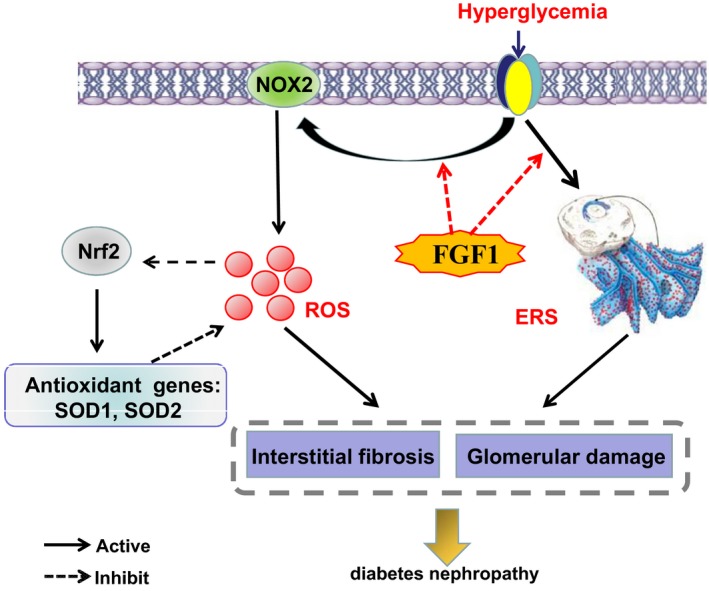
A Schematic showing the effect of cellular stress during FGF1 treatment for Diabetic nephropathy. FGF1 treatment blocked hyperglycemia‐induced oxidative stress though NOX2‐ROS‐Nrf2 signalling, and elevated ER stress in kidney, which ameliorated interstitial fibrosis and glomerular damage

In summary, our current study has further confirmed the protective role of FGF1 on DN with attenuation of renal fibrosis and glomerulosclerosis, and demonstrated that FGF1 administration blocked diabetes‐induced oxidative stress level though NOX2‐ROS‐Nrf2 signalling, and elevated ER stress (Figure 6), suggesting that reduction of cellular stress is another potential mechanism underlying FGF1 treatment for DN. These findings suggest that FGF1 also plays a potential renal protective effect for DN via exerting the effective glucose control function and subsequently reducing the cellular stress in kidney.

## COMPETING INTERESTS

The authors confirm that there are no conflicts of interest.
